# Massive Cerebrospinal Fluid Leak of the Temporal Bone

**DOI:** 10.1155/2016/7521798

**Published:** 2016-08-11

**Authors:** Giannicola Iannella, Alessandra Manno, Emanuela Pasqualitto, Andrea Ciofalo, Diletta Angeletti, Benedetta Pasquariello, Giuseppe Magliulo

**Affiliations:** ^1^“Organi di Senso” Department, University “la Sapienza”, Viale del Policlinico 151, 00161 Rome, Italy; ^2^“Radiology” Department, University “la Sapienza”, Viale del Policlinico 151, 00161 Rome, Italy

## Abstract

Cerebrospinal fluid (CSF) leakage of the temporal bone region is defined as abnormal communications between the subarachnoidal space and the air-containing spaces of the temporal bone. CSF leak remains one of the most frequent complications after VS surgery. Radiotherapy is considered a predisposing factor for development of temporal bone CSF leak because it may impair dural repair mechanisms, thus causing inadequate dural sealing. The authors describe the case of a 47-year-old man with a massive effusion of CSF which extended from the posterior and lateral skull base to the first cervical vertebrae; this complication appeared after a partial enucleation of a vestibular schwannoma (VS) with subsequent radiation treatment and second operation with total VS resection.

## 1. Introduction

Cerebrospinal fluid (CSF) leakage in the temporal bone region is defined as an abnormal communication between the subarachnoidal space and the air-containing spaces of the temporal bone [1, 2]. It may occur following brain and skull base surgery, temporal bone fractures, tumors, or congenital anomalies [3–5].

Some authors have suggested that radiotherapy (RT) may be considered a predisposing factor for development of temporal bone CSF leakage [2, 5–7]. In patients who have undergone presurgical radiotherapy, the likelihood of a successful dural repair is reduced due to the compromised dural and skull base blood supply, extensive scarring, and tissue hypoxia [5, 6, 8].

The authors present a rare case of a massive temporal bone CSF leak which occurred after surgery to partially enucleate a vestibular schwannoma (VS), subsequent radiotherapy, and further surgical treatment consisting of total VS resection.

## 2. Case Report

A 47-year-old Italian man came to our Department in January 2012 complaining of vertigo and headache. He showed a left facial nerve paralysis evaluated as grade V according to the House-Brackmann scale. He was surgically treated in 2009 by means of a partial intracapsular enucleation technique via a retrosigmoid approach for a left VS in another hospital. Postoperative adjuvant fractionated stereotactic radiotherapy (SRT) with a total dose of 40–50 Gy, administered in 20–25 fractions over a 5- to 6-week period, was performed.

**Figure 1:**
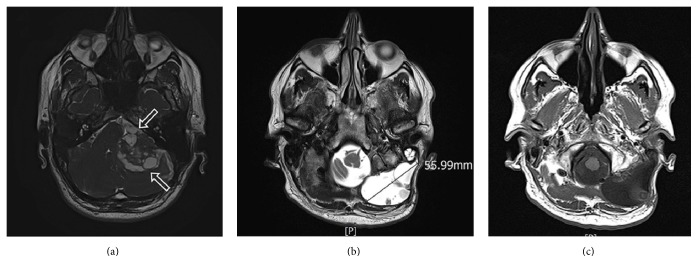
(a) Axial T2-weighed image. Cystic areas of CSF leak consequent to defect of dural repair (white open arrows). (b) Axial T2-weighed image. Hyperintense CSF leak measured 5.6 cm (arrow). (c) Axial T1-weighed image. Hypointense CSF leak.

Due to VS tumor regrowth, causing cerebellar compression and perilesional brainstem edema, the patient underwent a second neurosurgical operation by retrosigmoid approach in October 2011 with total VS removal. Preoperative ventriculoperitoneal shunt was placed in order to prevent a brain herniation. No apparent postoperative complications occurred; however, cystic areas of CSF leakage consequent to a defect of dural repair were visible on a subsequent control MRI (Figure 1(a)).

**Figure 2:**
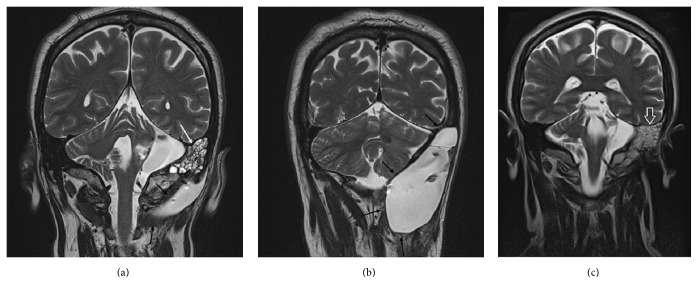
(a) Coronal T2-weighed image. Hyperintense CSF leak to the posterior skull base (black arrows). CSF leak in mastoid cells (white arrow). (b) Coronal T2-weighed image. Massive hyperintense CSF leak extended between the latera skull base and the fist cervical vertebra. Cerebellum compression visible (black arrows). (c) Postoperative coronal T2-weighed image. No CSF leak. Hyperintense signal to the mastoid air cells (white open arrow).

After 3 months of follow-up, the patient started to complain of worsening headache and severe dizziness. Moreover, CSF rhinorrhea was reported. Brain MRI with contrast medium performed in our Department showed a massive CSF leak which measured approximately 5.6 cm and 8.4 cm along its the transversal (Figure 1(b)) and longitudinal diameters, respectively. It was hyperintense on T2-weighted images (Figure 1(b)) and hypointense on T1-weighted sequences (Figure 1(c)). The CSF leak expanded from the posterior and lateral skull base to the first cervical vertebrae with dislocation of the sternocleidomastoid muscle (Figures 2(a) and 2(b)). CSF leakage in mastoid cells (Figure 2(a)) with cerebellar compression and dislocation was also evident (Figure 2(b)).

To treat the massive CSF leak, equine pericardium grafts were used to repair the posterior fossa dural defect and a subtotal petrosectomy [9–13] with blind sac closure of the external auditory canal, closure of the Eustachian tube, and obliteration of the middle ear and petrous bone was performed using abdominal fat.

After surgery, there was a marked improvement of vertiginous symptoms and headache. No new episodes of CSF rhinorrhea occurred.

At the most recent postoperative follow-up, 4-years after surgery, MRI showed no CSF leak recurrence and no parenchymal lesions (Figure 2(c)).

## 3. Discussion

Cerebrospinal fluid leakage in the temporal bone region is an uncommon condition with multiple potential etiologies [14]. It most frequently occurs in the context of traumatic temporal bone fracture and less often may arise spontaneously, secondary to erosive chronic otitis media, or as a result of surgical injury [2, 5, 15, 16]. To this regard CSF leakage remains one of the most frequent complications after VS surgery, despite the great advances made in surgical technique and experience, with an estimated incidence varying from 2 to 16% of cases [17–21].

Most dural defects can be easily managed during the initial operation; however, in some cases they may be up to 2 cm in diameter, cannot be sealed off spontaneously, or remain unrecognized until later [22–26].

This condition usually presents with nonspecific symptoms such as aural fullness, tinnitus, headache, and vertigo. Rhinorrhea, otorrhea, or middle ear effusion could, in certain cases, camouflage CSF leakage [6, 13, 27].

We describe the case of a 47-year-old man with a massive effusion of CSF extending from the posterior and lateral skull base to the first cervical vertebrae, which appeared after partial enucleation of a VS with subsequent radiation treatment and a second operation consisting of a total VS resection.

Imaging studies are essential for determining the site of the CSF leak in the temporal bone. High-resolution noncontrast CT (HRCT) is considered the best initial diagnostic study. HRCT usually identifies any bony defects of the temporal bone but might not demonstrate the site of the dural tear [2, 13, 18, 27].

MRI is a sensitive and accurate technique for detection of CSF leaks which usually appear hyperintense on T2-weighted images and hypointense on T1-weighted sequences: it also offers excellent anatomical detail without risk of radiation [1, 2, 26].

In the differential diagnosis of temporal bone CSF collections, subdural hygroma, meningocele, and arachnoid cyst should all be considered. Magnetic resonance imaging is able to distinguish isolated temporal bone CSF leakage from middle ear/mastoid encephalocele because the CSF signal is usually bright on T-2 weighted images, and the encephalocele appears contiguous with the brain [1, 2, 13, 18, 26]. Arachnoid cysts are isointense while neoplastic, hemorrhagic, or inflammatory cysts are hyperintense in comparison to CSF on T-1 sequences [13, 28].

Important risk factors for a postoperative CSF leak after VS surgery do exist [5, 21]. In this context patient age, sex, body mass index (BMI), tumor size, tumor side, history of prior tumor treatment, operative duration, surgical approach, extent of resection, and extensive dural damage have been the most studied risk factors [5, 18, 19, 21]. Copeland et al. [21], in a systematic review, analyzed 457 patients surgically treated for VS and found that an elevated BMI is a sure risk factor for the development of a postoperative CSF leak. Patients undergoing a translabyrinthine approach or having longer operative times also have an increased risk of developing a postoperative CSF leak [21, 22].

In addition to the above some authors have claimed that an important risk factor related to CSF leak formation seems to be adjuvant RT [2, 6, 7, 27].

Radiotherapy is considered a predisposing factor to temporal bone CSF leak development because it can impair the dural repair mechanisms, causing an insufficient dural sealing [5, 7]. After radiotherapy, the likelihood of a successful repair is reduced due to the compromised blood supply to the dura and skull base, extensive scarring, and tissue hypoxia [2, 17, 27]. In this regard, Gerganov et al. [6] found that the risk of CSF leakage in patients treated with surgery and radiotherapy is certainly worse than in patients treated with radiosurgery alone.

Recognizing CSF risk factors can allow surgeons to better counsel patients regarding the risks of VS surgery and potentially modify perioperative management in order to reduce the risk of developing a postoperative CSF leak [5, 19].

Subtotal petrosectomy with obliteration of the middle ear cleft seems to be an efficient solution for stubborn CSF otorrhea [2, 12, 13, 15, 29, 30]. Kronenberg et al. [18] asserted that in 44% of cases CSF leakage was through the internal auditory canal, making it advisable to extend the subtotal petrosectomy technique to the inner ear and the internal auditory canal in order to seal any possible leakage from this point. A reliable result can be achieved only by total exenteration of the temporal bone cavities, the semicircular canal, vestibulum, and cochlea [18, 30].

In patients who must be submitted to subtotal petrosectomy, a preoperative high-resolution CT scan is mandatory [2, 13, 18]. If patients have had previous surgery, it is particularly important in order to prevent complications involving the temporal bone structures which may have been exposed at primary surgery, for example, facial nerve, sigmoid sinus, dura, and mastoid cells [2, 13, 18].

We believe that in patients submitted to radiotherapy, dural repair after new surgical treatment may be less efficient with the possibility of subsequent massive CSF leakage. In the light of the increasingly important roles played today by RT in the definitive or adjuvant management of vestibular schwannomas, this aspect should not be underestimated. Therefore, surgical reconstruction of the skull base dura should be done more carefully in patients who previously underwent RT. In the event of massive CSF leakage, it is mandatory not to delay surgical obliteration of all accessible pneumatized spaces in the petrous bone, in order to close all possible fistulas and to prevent meningitis. Our therapeutic strategy consisting of subtotal petrosectomy, demonstrated good therapeutic efficacy with improvement of clinical symptoms and a low risk of CFS leak recurrence even after a long follow-up.
